# Hospital for Sick Children

**Published:** 1902-06-07

**Authors:** 


### HOSPITAL FOR SICK CHILDREN.

### NEW OUT-PATIENTS' DEPARTMENT WANTED.

The Duke of Fife, President of the Hospital for Sick Children, Great Ormond Street, took the chair at the annual Court on Wednesday, May 28th, and was well supported by the committee and some fifteen or sixteen governors, two or three of whom were ladies. The meeting, the chairman said, was for business, but as this was the fiftieth annual Court, he might be allowed to look back on the history of what was not only the oldest but the largest children's hospital in the British Empire. It was opened with 20 beds in January 1852, under the immediate patronage of her late Majesty Queen Victoria, who subscribed £100. During the first year 143 in-patients and 1,250 out-patients were cared for; and comparing these figures with those of 1901, it would be seen that the increase had been enormous. In 1901 the figures were: In-patients, 2,300; new out-patients, 25,000; while there was a daily average attendance of nearly 350 throughout the year of 306 working days. Over three-quarters of a million children had been treated since the beginning. As the work had increased, so had the cost, and personally he deplored some of the means adopted for the raising of the necessary funds. They were, however, unavoidable, until the remarkable and optimistic prophecy of Sir Henry Burdett, as set forth in a letter to the *Times*, was fulfilled. If Sir Henry Burdett could promise this hospital £70,000 a year, or even a couple of hundred thousand down, it would remove all financial anxieties. He concluded his speech with a warm appreciation of the work of Mr. Arthur Lucas and Mr. John Murray on the committee, and of the indefatigable services as secretary of Mr. Adrian Hope, to whose unfailing courtesy during the twelve years of his presidency he was glad to be able to bear record.

Sir William Agnew, in proposing the adoption of the minutes of the last annual meeting, said he hoped next year there would be a better financial statement; he would give a handsome yearly subscription himself as a beginning.

Mr. Arthur Lucas, in moving the adoption of the report, said that the success of the committee's work was in the main due to the fact that everyone in the hospital, from the highest surgeon down to the office-boy, could bring them their grievances and have them adjusted. The help given by the medical staff was invaluable, and had raised the hospital to its present high position. The committee was a very hardworking one.

#### The Out-Patients' Department.

Sir Henry Burdett, who seconded the motion, said he welcomed the presence of His Grace the Duke of Fife, because he knew that the increased strength, efficiency, and usefulness of a great hospital like this was due to the circumstance that individuals in high positions, and those in humble ones also, recognised the fact that they owed a measure of personal service. It was perfectly clear to him that if the hospitals were to take their rightful place, those who occupied the position of president must be useful and not merely ornamental. Turning to the report, the speaker said that Mr. Hope, of whose work he had some knowledge, showed signal ability in making his points clear; at the end of the book he had given a statement of the hospital's needs.

"It strikes home," Sir Henry Burdett continued, "as something remarkable, that those needs do not include a new out-patients' department. I came here as a visitor for the Prince of Wales's (now King Edward's) Fund, and I did wish that you, your grace, as President, and the committee, would make it your business to attend at the busiest time in the morning."

Several voices said "We have"; and the speaker went on If they did that, he felt sure they would not rest content until they had a new out-patients' department, or another room elsewhere to take off some of the patients. At the present time, if they could not get the money to build a new department, they could at least reduce the numbers, and he believed that step would be followed by the necessary funds being forthcoming. He hoped they would forgive him for emphasising the point, because it was a sad thing that they should have their out-patients' department in its present condition, and it could not go on. Personally, he would do anything in his power for them, but he did hope the committee would look into it, and that they would commence their investigations by a personal visit.

#### How the Coronation Gift would Help.

Proceeding to comment on the remark made by the chairman about his letter to the *Times*, Sir Henry Burdett said his hope of seeing the London hospitals freed from debt was based on this—that if the King's wish were fulfilled, and King Edward's Fund had a yearly income of £100,000, it would be an accomplished fact. The Fund already had £70,000 a year, and other sums were in prospect, and it rested with the country whether the £50,000 more would be forthcoming by Coronation Day. He had been speaking on the subject in country villages; and—as he thought he detected a frown on the face of the President—he wished to say that whatever claims other hospitals had, that of the London hospitals was also very great, and there was no part of the country of which this was not true. London was the last resort of the sick; it was the centre of scientific developments, and, further, it would be impossible for those in many parts of the country to have the medical care they needed, were it not for the training received at the London hospitals. From his own observations at the meetings he had attended, the last letter of Sir Francis Knowles had gone home to the hearts of the people, and if the revenue of the fund were increased as he hoped, the revenue of this hospital, among others, would also be increased—enough, he hoped, to bring it up to the necessary standard of efficiency. He was one who felt that the annual meeting of a hospital was the time to press home, through the press, the hospital's claims on all people. He was old enough in hospital work to remember that there was a great controversy when this hospital set up a department for dealing with little children, but events had proved that its claims were more than justified.

Mr. Justice Kekewich said the committee was not content with the out-patients' department, and would not rest until they had a new one; they thought, however, after full discussion, that it would be extremely wrong to embark on an expenditure of something like £30,000 when they had not got the money. "Find us that money," he added, "and we will do it."

